# Iris Avulsion During Corneal Wound Hydration After Uneventful Cataract Extraction

**DOI:** 10.7759/cureus.74807

**Published:** 2024-11-30

**Authors:** Lampros Lamprogiannis, Padmanabha P Syam, Arsh Patel, Kaushal Pillai Syam, George Voyatzis

**Affiliations:** 1 Ophthalmology, Ophthalmica Institute, Thessaloniki, GRC; 2 Ophthalmology, Anglia Community Eye Services, Peterborough, GBR; 3 Ophthalmology, Peterborough City Hospital, Peterborough, GBR; 4 Medicine, Imperial College London, London, GBR; 5 Ophthalmology, Moorfields Eye Hospital, London, GBR

**Keywords:** cataract, complication, iris avulsion, phacoemulsification, wound hydration

## Abstract

We report a case of spontaneous total iris avulsion that occurred during corneal wound hydration following an uneventful phacoemulsification procedure. An 86-year-old woman underwent cataract surgery on her right eye, during which a single-piece acrylic intraocular lens (Bausch and Lomb Akreos Adapt Advanced Optics, Bausch and Lomb Incorporated, Rochester, NY, USA) was implanted in the bag, and the viscoelastic material was removed. During corneal wound hydration, total iris avulsion was observed, with the iris prolapsing out of the anterior chamber through the main wound. Wound hydration was completed, and no additional surgical intervention was performed. Four months postoperatively, the patient achieved a best corrected visual acuity of 20/50. This case highlights the importance of recognizing the pressure dynamics during corneal wound hydration and the potential risk of complications.

## Introduction

Iridodialysis, defined as the avulsion of the iris from the ciliary body, is most commonly caused by ocular trauma [1]. Iatrogenic iridodialysis is a recognized complication of intraocular surgery [2-4], typically resulting from direct intraoperative injury to the iris. Studies [5-7] have shown that the pressure generated during corneal wound hydration in cataract surgery can result in severe complications, such as Descemet’s membrane detachment and ejection of the cannula. However, to the best of our knowledge, no prior reports have documented an association between expulsive iridodialysis and corneal wound hydration during intraocular surgery.

## Case presentation

An 86-year-old woman presented to the cataract clinic at Peterborough City Hospital with a two-year history of decreased vision in her right eye. Examination revealed a moderate nuclear sclerotic cataract. She had undergone uneventful cataract extraction in the left eye at our department the previous year. There was no history of other ocular pathology or prior ocular trauma. Her corrected distance visual acuity (CDVA) (logMAR) was 0.4 in the right eye and 0.1 in the left eye. After preoperative assessment, the patient was scheduled for cataract extraction.

**Figure 1:**
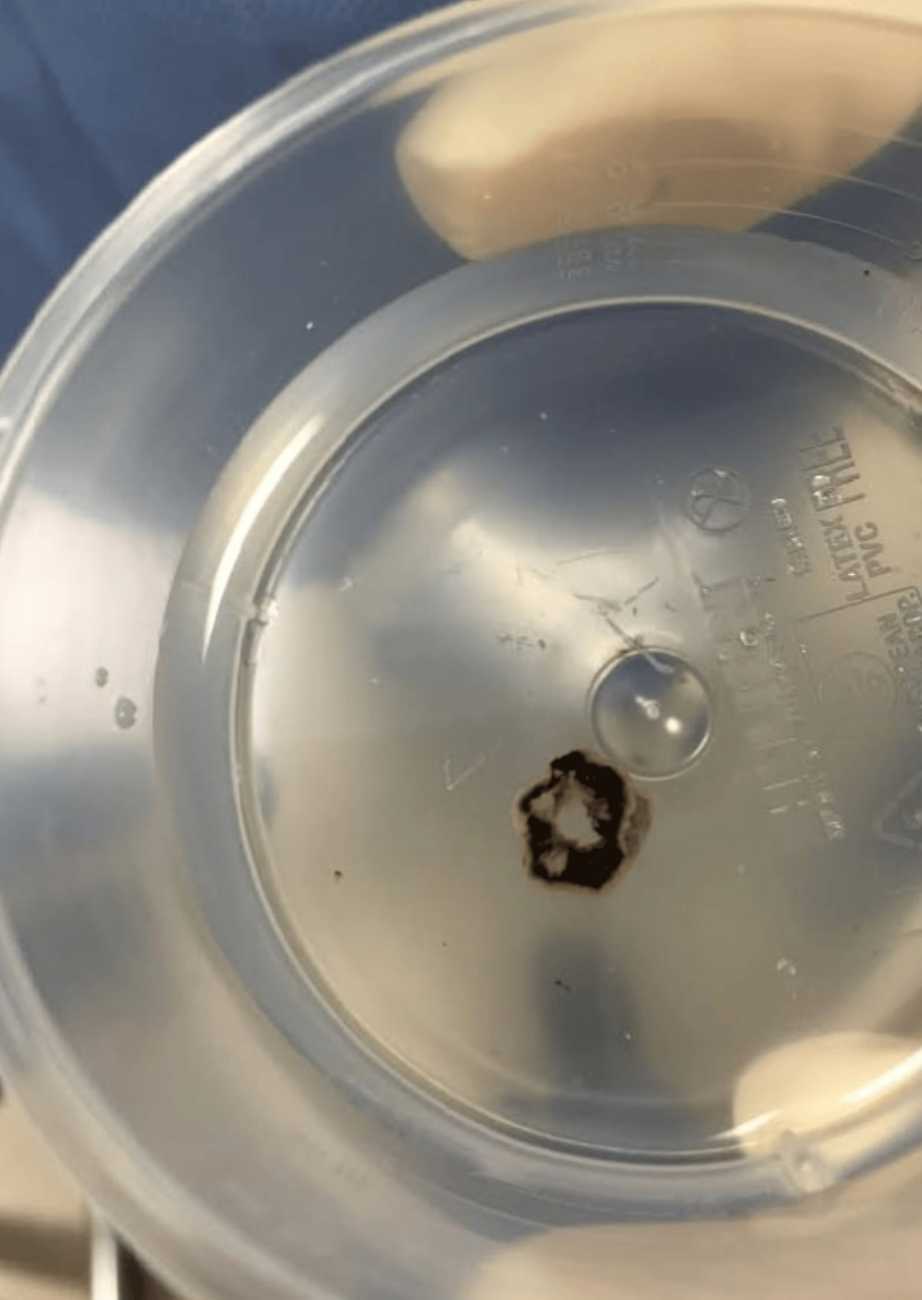
The avulsed iris, removed after prolapsing through the main incision, displayed in a plastic container

A temporal approach was used under sub-Tenon’s anesthesia with a Stellaris phacoemulsification machine (Bausch & Lomb) and a 2.85 mm temporal clear corneal incision. The cataract was removed without complications, and a three-piece acrylic intraocular lens (Bausch and Lomb Akreos Adapt Advanced Optics, Bausch and Lomb Incorporated, Rochester, NY, USA) was implanted in the bag. During the procedure, the iris prolapsed through the main wound. After removing the remaining viscoelastic, corneal wound hydration was performed. During this step, a total expulsive avulsion of the iris was observed, with the iris exiting the anterior chamber through the main wound (Figure 1).

Subsequently, the anterior chamber was irrigated and aspirated. Intracameral cefuroxime 1.0 mg (Aprokam) and subconjunctival betamethasone were administered.

On the first postoperative day, the patient reported blurred vision and glare. Examination revealed moderate stromal corneal edema, microscopic hyphema, and anterior chamber inflammation. The uncorrected distance visual acuity was count fingers. Postoperative treatment included iopidine 0.5% eye drops and a combination of topical dexamethasone, neomycin, and polymyxin B eye drops.

By six weeks postoperatively, significant improvement was observed. The patient was satisfied with her vision, and glare no longer significantly affected her quality of life. No inflammation was present, and intraocular pressure (IOP) was within normal limits at 15 mmHg. The CDVA (logMAR) was 0.3, improving to 0.2 with a pinhole. Close observation was deemed the appropriate management, as the postoperative aniridia did not result in reduced vision, and the patient expressed satisfaction with the outcome.

At the most recent follow-up, four months after the operation, vision remained stable, and the patient continued to be pleased with the results.

## Discussion

Hydration of corneal wounds is widely employed by ocular surgeons as an effective method for sealing surgical incisions [8]. However, it is associated with potential intraoperative complications. A recent study [9] demonstrated that the pressure generated during stromal wound hydration can exceed the threshold required to eject a loose cannula, potentially leading to cannula-related ocular injuries. Our case illustrates that such pressure levels may also result in iridodialysis, particularly in patients predisposed to zonular weakness, such as those of advanced age or with pseudoexfoliation syndrome.

The exact mechanism of this complication remains unclear, but it is known that hydration causes a transient rise in IOP. When the main surgical wound reopened, the abrupt release of fluid may have dragged the iris entirely out of the anterior chamber. Similar cases of iris expulsion have been reported following acute IOP spikes caused by blunt trauma [10] or vigorous vomiting due to the Valsalva maneuver [11]. These parallels suggest that elevated intraoperative IOP might play a critical role in such severe complications.

It is also plausible that the patient exhibited mild intraoperative floppy iris syndrome (IFIS), as the iris prolapse occurred without associated miosis. Advanced age was the only apparent risk factor for IFIS in this case, as the patient was not using alpha-1 adrenergic receptor antagonists [12]. Additionally, manipulation of the prolapsed iris during surgery could have contributed to its damage, increasing the likelihood of subsequent avulsion. Factors such as mechanical contact of the iris with surgical instruments, fluid turbulence, and ultrasound energy in the anterior chamber are known to exacerbate iris chaffing and increase the risk of complications, including iris avulsion [13].

Postoperative aniridia can significantly affect visual outcomes, causing symptoms such as glare, photophobia, and reduced vision, potentially necessitating artificial iris implantation to restore function and comfort [14].

## Conclusions

Adequate precautions are essential when risk factors for IFIS are identified or if iris prolapse occurs during surgery. These precautions include minimizing iris manipulation, using low ultrasound energy, and adhering to meticulous surgical techniques to avoid unnecessary contact with the iris. Additionally, intraoperative spikes in IOP should be prevented. In cases where the risks associated with wound hydration may surpass its benefits for sealing incisions, it is reasonable to omit hydration altogether.
